# Examining the Neural Markers of Speech Rhythm in Silent Reading Using Mass Univariate Statistics of EEG Single Trials

**DOI:** 10.3390/brainsci14111142

**Published:** 2024-11-14

**Authors:** Stephanie J. Powell, Srishti Nayak, Cyrille L. Magne

**Affiliations:** 1 Interdisciplinary Ph.D. Program in Literacy Studies, Middle Tennessee State University, Murfreesboro, TN 37132, USA; stephanie.wolfe@mtsu.edu; 2 Department of Otolaryngology-Head and Neck Surgery, Vanderbilt University Medical Center, Nashville, TN 37232, USA; srishti.nayak@vumc.org; 3 Vanderbilt Genetics Institute, Vanderbilt University Medical Center, Nashville, TN 37232, USA; 4 Psychology Department, Middle Tennessee State University, Murfreesboro, TN 37132, USA

**Keywords:** speech rhythm, prosody, implicit prosody, lexical stress, silent reading, EEG, ERP, neural oscillations

## Abstract

Background/Objectives: The Implicit Prosody Hypothesis (IPH) posits that individuals generate internal prosodic representations during silent reading, mirroring those produced in spoken language. While converging behavioral evidence supports the IPH, the underlying neurocognitive mechanisms remain largely unknown. Therefore, this study investigated the neurophysiological markers of sensitivity to speech rhythm cues during silent word reading. Methods: EEGs were recorded while participants silently read four-word sequences, each composed of either trochaic words (stressed on the first syllable) or iambic words (stressed on the second syllable). Each sequence was followed by a target word that was either metrically congruent or incongruent with the preceding rhythmic pattern. To investigate the effects of metrical expectancy and lexical stress type, we examined single-trial event-related potentials (ERPs) and time–frequency representations (TFRs) time-locked to target words. Results: The results showed significant differences based on the stress pattern expectancy and type. Specifically, words that carried unexpected stress elicited larger ERP negativities between 240 and 628 ms after the word onset. Furthermore, different frequency bands were sensitive to distinct aspects of the rhythmic structure in language. Alpha activity tracked the rhythmic expectations, and theta and beta activities were sensitive to both the expected rhythms and specific locations of the stressed syllables. Conclusions: The findings clarify neurocognitive mechanisms of phonological and lexical mental representations during silent reading using a conservative data-driven approach. Similarity with neural response patterns previously reported for spoken language contexts suggests shared neural networks for implicit and explicit speech rhythm processing, further supporting the IPH and emphasizing the centrality of prosody in reading.

## 1. Introduction

The rhythmic flow of spoken English is shaped by the patterned distribution of lexical stress, i.e., the relative prominence of syllables within words. By emphasizing specific syllables with greater intensity, length, and pitch, this stress pattern forms a metrical structure that guides speech segmentation and comprehension. In English, lexical stress is unevenly distributed across content words, with a strong bias toward first-syllable stress (85–90%) [1]. Through statistical learning, this uneven distribution is leveraged by English-learning infants who show a preference for words with first-syllable stress and acquire them earlier in language development than words containing second-syllable stress [2,3,4]. The location of stressed syllables serves as a powerful cue for word segmentation in the continuous stream of speech. Both infants and adults use this cue to identify word boundaries, thereby facilitating word learning [5,6,7,8]. Furthermore, the ability to perceive, understand, and reproduce the patterns of stress in spoken language (i.e., speech rhythm awareness) may facilitate the acquisition of essential reading skills by acting as a bridge between oral language and written language [9].

While English exhibits a rhythmic pattern that tends toward isochrony, where the timing between stressed syllables is equal [10,11], it does not rigidly adhere to this principle. Research showed that while the intervals between stressed syllables tend to be more consistent in carefully articulated speech or in poetry, where rhythmic patterns are deliberately emphasized, the timing can be more flexible and less predictable in casual conversation [12,13]. Yet, listeners have been found to prefer contexts with regular rhythm [14], and such contexts have been associated with improved processing of phonological information [15,16], syllables [17], semantic information [18], and syntax [19], ultimately leading to enhanced sentence comprehension [18].

### 1.1. Neural Correlates of Speech Rhythm

Complementary insights into the neural mechanisms underlying lexical stress perception and speech rhythm expectancy have been provided by event-related potentials (ERPs) and time–frequency representations (TFRs) derived from electroencephalographic (EEG) data. ERPs offer high temporal resolution, precisely tracking neural events as they unfold over time in response to a stimulus, while TFRs reveal the brain’s parallel processing of information, showing multiple neural processes co-occurring and interacting across distinct frequency bands.

Research that utilized ERPs to examine neural responses to unexpected rhythms in spoken language generally produced consistent findings. For example, studies that utilized the violations of rhythm and/or metrical structures [18,20,21,22,23,24], words with correct but unexpected rhythmic/metrical patterns [25,26,27], or pseudowords with unexpected stress patterns [28] found increased negativity in the first 400 ms post-stimulus onset for rhythmically anomalous or unexpected words, though some found negativities up to 1000 ms post-stimulus onset [20,23,26]. These negativities are sometimes followed by late positivities between 500 and 900 ms [18,20,21,22,23,24,26], which some attribute to task-relevant processing [20,21].

TFR analyses provide a window into neural oscillations—the rhythmic fluctuation in brain activity occurring at distinct frequencies. Converging data suggest that theta band oscillations (4–8 Hz) are particularly crucial for extracting prosodic cues related to lexical stress and speech intelligibility [29]. Additionally, theta oscillations are thought to regulate higher-frequency activity, such as gamma oscillations (>30 Hz). It was proposed that this theta–gamma cross-frequency coupling ensures that gamma oscillations encode phonemic details in sync with speech rhythm at the syllabic level, as dictated by theta oscillations [30]. Furthermore, research suggests potential roles for alpha (8–13 Hz) and beta (13–30 Hz) oscillations in speech rhythm perception. Alpha oscillations have been linked to attentional demand [31] and might be involved in the processing of stressed syllables, which act as attentional anchors within the speech stream, thus aiding in parsing and interpreting incoming linguistic information [15]. Beta oscillations, on the other hand, are thought to be involved in top-down control mechanisms and may contribute to the temporal prediction and anticipation of upcoming speech units [32]. Overall, these findings thus underscore the complexity of the neural dynamics underlying speech rhythm perception and highlight the importance of investigating the contributions of different frequency bands to this process.

### 1.2. Implicit Prosody

Although traditionally linked to spoken language, prosody has emerged as a potential factor influencing reading abilities, with mounting evidence suggesting a correlation between prosodic sensitivity and individual reading variations. This relationship is further supported by the Implicit Prosody Hypothesis (IPH). The IPH, proposed by Bader [33] and Fodor [34], posits that during silent reading, individuals internally generate prosodic representations that mirror the explicit prosody used in similar spoken contexts. In essence, even when reading silently, we unconsciously apply the rhythms, stresses, and intonations we would use if speaking the text aloud. This hypothesis is supported by behavioral studies that demonstrated that many features of explicit prosody are evident during silent reading, influencing how we comprehend phrases and sentences [33,35]. The significance of prosody in silent reading extends beyond general comprehension. Research on lexical stress, for instance, suggests it may be an inherent component of word processing. Using eye-tracking, ref. [36] found readers spent more time reading polysyllabic words with two stressed syllables, regardless of the word length or frequency. Moreover, readers generate expectations for prosodic representations of words while reading silently. Noun–verb homographs are words containing identical spellings but pronunciations that vary by stress pattern according to their grammatical function (e.g., ABstract as a noun vs. abSTRACT as a verb, where the capital letters denote the stressed syllable). The researchers embedded these words in syntactically ambiguous sentences and found readers were slower to read the target words when ambiguity resolution required a shift in stress than when it did not [37,38]. Furthermore, evidence suggests that internally generated speech rhythm plays a role in the early stages of sentence parsing. When readers were presented with sentences that contained ambiguous noun–verb homographs differentiated by lexical stress, they initially assigned stress to maintain the rhythm established by the preceding words in the sentence. It was only when the complete sentence was available to readers that the syntactic information overrode their initial stress assignment for ambiguous words [39].

While a growing body of behavioral research supports implicit rhythm sensitivity during silent reading, the underlying neural mechanisms remain relatively unexplored compared with the body of research on explicit rhythm sensitivity. The few existing studies present divergent findings. For example, in a pilot study on eight participants, Magne and colleagues [40] visually presented sequences of English words that all contained the same stress pattern, either all first-syllable stressed words (i.e., trochaic) or all second-syllable stressed words (i.e., iambic), followed by target words that either matched or mismatched the stress pattern of the preceding sequence. They found increased negativities in the N400 time window for both iambic and trochaic target words when they were unexpected, with larger effects for trochaic targets.

In a similar study with a larger sample, Kriukova and Mani [41] recorded neural responses while participants read sequences of Dutch disyllabic words. The sequences consisted of either three trochaic words or three iambic words, followed by target words that were metrically consistent or inconsistent with the prime sequences. They did not observe a significant negativity for inconsistent trochaic words, despite a small, non-significant trend in that direction (see Figure 1B in [41]). Furthermore, they reported an increased positivity for iambic words. Fotidzis and colleagues [42] examined neural responses to silently read words that were presented following an auditory rhythmic tone prime that either matched or mismatched the rhythmic structure of the targets. They found increased negativities between 300 and 708 ms post-stimulus for words that did not match the rhythm structure of the auditory primes.

Finally, Breen and colleagues [43] embedded stress-alternating noun–verb homographs (e.g., PERmit vs. perMIT) in rhyming couplets with a regular trochaic or iambic metric structure. The homograph word class was either consistent or inconsistent with the stress expectation generated by the couplets. They observed increased early (80–155 ms) and late (325–375 ms) negativities for inconsistent trochaic words and, similar to [41], increased positivities (365–435 ms) post-stimulus for inconsistent iambic words. In sum, not only do the results of these studies differ from each other, but they also diverged from the spoken language literature in that previous auditory studies have generally found enhanced negativities between 300 and 500 ms post-stimulus for unexpected lexical stress compared with expected lexical stress [17,26,27,28]. There are many differences between these studies, and though this is also the case for the auditory studies of lexical stress, it is possible that neural responses to implicit lexical stress are more subtle and harder to detect. Further research is warranted to determine factors that contributed to these divergent findings.

### 1.3. Design of Present Study and Predictions

The goal of the present study was to identify the differences in electrophysiological responses when the lexical stress of targets aligned or deviated from the expected rhythmic pattern. Previous research revealed several distinctions related to the use and processing of trochaic and iambic words. These include a higher prevalence of trochaic than iambic words in English [1], a developmental trajectory where trochaic words are typically acquired earlier than iambic words [2,3,4], and more varied ERP responses to unexpected iambic compared with unexpected trochaic words [40,41,42,43]. Considering these findings, we also sought to explore whether the brain responses differed between trochaic and iambic words during silent reading.

To this end, we manipulated the stress pattern and metrical expectancy of words in a silent reading task. EEGs were recorded while participants silently read five-word sequences. The first four words of each sequence were either all trochaic or all iambic, while the stress pattern of the fifth word in the sequences either matched or did not match the stress pattern of the previous four words. Both the ERPs and time–frequency characteristics of the EEG data were analyzed to identify the neural correlates of implicit stress sensitivity. Building on previous ERP research that manipulated overt [20,21,27,28] or implicit [40,42,43] speech rhythm cues, we hypothesized increased negativity in the 200–600 ms window following unexpected trochaic patterns. Given the less consistent findings for iambic words, we predicted either a similar but attenuated negativity or potentially an increased positivity for unexpected iambic patterns.

In the time–frequency domain, we proposed that the stress pattern expectancy would primarily modulate neural activity in the theta band. More specifically, we anticipated an increased theta activity for expected stress patterns, reflecting enhanced neural synchronization with predictable word metrical structure. This hypothesis was grounded in previous research that highlighted the role of theta oscillations in encoding and integrating rhythmic information in speech [29]. Alternatively, given that theta activity increase was also observed for semantically unexpected words, reflecting the brain’s engagement in control processes [44], we hypothesized that an increased beta activity may be observed for unexpected stress patterns if they disrupt lexical access, as suggested in previous research [21,22]. Interestingly, these predictions align with the ongoing debate regarding the functional significance of negative ERPs associated with rhythmically unexpected words, potentially supporting the interpretations of these either reflecting semantic processing difficulty and/or rule-based/predictive sequencing error detection [28]. Note these potential outcomes are not mutually exclusive, as distinct brain sources have been implicated in theta activity related to the rhythmic and semantic aspects of speech, suggesting different neural substrates for expected and unexpected stress patterns.

## 2. Materials and Methods

### 2.1. Participants

Twenty college students received course credit for their participation (11 females and 9 males, mean age = 22, SD = 3, age range: 18–28). Data from two participants were ultimately excluded from the analysis due to excessive artifacts in the EEG, leaving 18 participants included in the final analysis. All participants were right-handed (assessed by the Edinburgh Handedness Inventory [45]), had normal hearing and vision, and were native speakers of English. Written consent was obtained from each participant prior to their participation in the experiment. Approval for this study was granted by the Institutional Review Board of Middle Tennessee State University.

### 2.2. Stimuli

Four types of word sequences varying in stress pattern type and/or metrical expectancy were constructed using bisyllabic nouns and adjectives selected from the English Lexicon Project database [46]. We focused on bisyllabic words because they allowed for straightforward manipulation of the stress patterns (strong–weak vs. weak–strong) while minimizing the influence of confounding linguistic factors, like syntax and morphology, which are more prevalent in longer words. For example, “doctor” (strong–weak) and “balloon” (weak–strong) provide a clear contrast for examining stress perception. By contrast, stress placement in longer words is often influenced by etymology and affixation (e.g., “A-cid” vs. “a-CID-ic”), introducing potential confounding variables. Furthermore, compared with longer words, bisyllabic words reduce the cognitive load on participants, ensuring that the observed effects are primarily attributable to metrical regularity and not working memory limitations.

A total of 500 words were selected from the database, with half (250) being trochaic (i.e., containing first-syllable stress), and half (250) being iambic (i.e., containing second-syllable stress). These words were then used to build 100 five-word sequences in which the initial four words followed either a trochaic or iambic stress pattern. Metrical expectancy was manipulated by varying the stress pattern of the fifth word (target word) of each sequence. In metrically expected conditions, the stress pattern matched that of the first four words, whereas in metrically unexpected conditions, the stress pattern differed from the preceding four. A total of twenty-five sequences were created for each of the four conditions (trochaic expected, iambic expected, trochaic unexpected, and iambic unexpected). Two counterbalanced lists of the 100 word sequences were used to ensure that the target words were presented in both expected and unexpected conditions across participants, with each participant seeing each target word only once. Examples of stimuli in each experimental condition are presented in Table 1.

The lexical frequencies of the individual words within each sequence was controlled using the Hyperspace Analogue to Language (HAL) frequency norms [47] to ensure equivalent frequencies between the initial four words and the target word (see Table 2). To minimize semantic relatedness, words within each sequence were evaluated by two independent judges unaware of the purpose of the experiment. Additionally, the web-based Latent Semantic Analysis (LSA) tool was used to gauge semantic similarity between the target word and the preceding four words in the sequence. The LSA generates a similarity score ranging from 0 (indicating no relation) to 1 (indicating a strong relation). The LSA values indicate that there were low semantic similarities for metrically expected (mean = 0.16, SD = 0.12) and unexpected conditions (mean = 0.12, SD = 0.09).

### 2.3. Procedure

**Figure 1:**
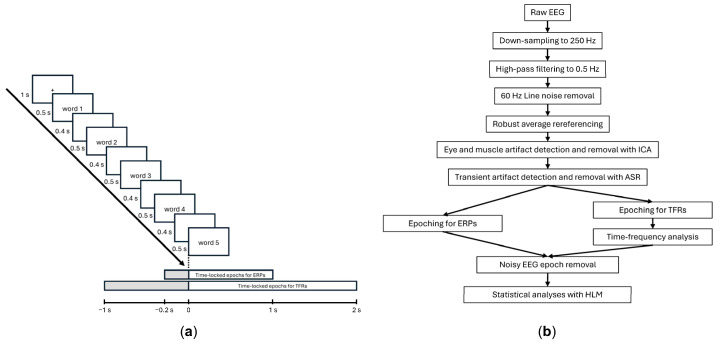
Experimental paradigm and EEG preprocessing: (**a**) Presentation of the visual stimuli over time within each trial (presented from top left to bottom right); (**b**) Schematic overview of the EEG data-processing pipeline (see Section 2 for details on each processing step).

Participants were seated in a soundproofed and electronically shielded room. They were positioned approximately one meter in front of a computer monitor. Stimuli were presented in black lowercase letters on a white background using E-prime 2.0 Professional with Network Timing Protocol (Psychology Software Tools, Inc., Pittsburgh, PA, USA). Each word sequence was presented one word at a time for 0.5 s, followed by a blank screen for 0.4 s. A fixation cross was displayed in between word sequences for 1 s (Figure 1a).

Participants viewed a total of 100 unique word sequences broken down into two blocks of 50 word sequences each. The order of trials was randomized within each block, and the order of blocks was counterbalanced across participants. To maintain the participants’ attention to the stimuli, they were told they were participating in a memory task and should carefully read the word sequences. They were not informed about the manipulation of stress patterns within the sequences. The entire experimental session lasted one hour.

### 2.4. EEG Acquisition and Preprocessing

Continuous EEG data were recorded at a sampling rate of 500 Hz on a MacBook Pro computer using a 64-channel Hydrocel Geodesic Sensor Net (EGI, Eugene, OR, USA) connected to a Net Amps 300 amplifier (Electrical Geodesics, Inc., Eugene, OR, USA). Electrode impedances were kept below 50 kΩ, and data were referenced online to Cz. Data preprocessing (Figure 1b) was performed in MATLAB R2024a (The MathWorks, Inc., Natick, MA, USA) using the EEGLAB toolbox [48]. The raw signal was first down-sampled to 250 Hz for computational efficiency and high-pass filtered at 0.5 Hz. We used the PREP pipeline plugin [49] to detect bad channels, apply a robust re-reference, and remove the 60 Hz line noise. Next, we performed an independent component analysis (ICA) using the runica algorithm on a copy of the data that was down-sampled to 100 Hz and high-pass filtered at 2 Hz to improve the efficiency of the ICA decomposition. The resulting ICA weights were then applied to the original data. The ICLabel function was used to automatically label each component. Any component labeled as indicating eye movements or muscle activity with a probability exceeding 90% was excluded. Finally, we used the artifact subspace reconstruction (ASR) algorithm and a 20-burst detection criteria threshold [50] to automatically identify and remove portions of the EEG containing large transient artifacts.

### 2.5. Statistical Analysis

To analyze the single-trial ERPs elicited by the critical word (i.e., the fifth word of each sequence), EEG epochs were extracted from −0.2 to 1 s relative to the word onset, and epochs were visually inspected and discarded if any remaining artifacts were present. A baseline correction was performed by averaging data from 200 ms to 0 ms pre-stimulus onset and subtracting this average from the rest of the time points. The TFRs were computed on epochs from −1 to 2 s relative to the critical word. Each epoch was convolved with a Hanning-windowed sinusoidal Morlet wavelet. A total of 13 log-spaced frequencies that ranged from 4 Hz to 30 Hz were calculated every 4 ms with an increasing number of wavelet cycles of 3 at 4 Hz and 11.25 at 30 Hz. The maximum wavelet window length was 836 ms at 4 Hz.

**Figure 2:**
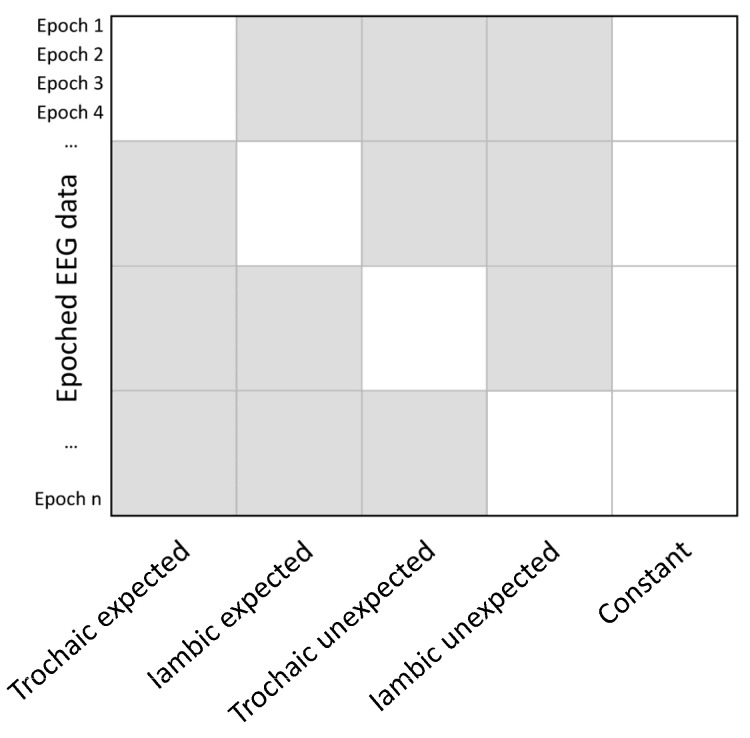
Design matrix. The design matrix X encoded the values of the predictor variables (i.e., the four combinations of stress expectancy and stress pattern) for each trial. A separate regression model was fit at each time point and electrode (and frequency for TFRs) using the EEG measured across epochs as the dependent variable. The resulting beta coefficients, representing the effect of each predictor, were then subjected to a 2 × 2 ANOVA.

We implemented hierarchical linear modeling with the LIMO EEG plug-in for EEGLAB [51]. To account for the within-subject variability across single trials, a general linear model was first implemented to estimate the beta parameters that represented the effect of each experimental condition (i.e., the four combinations of expectancy and stress pattern) using an ordinary least squares (OLS) regression. The within-subject model of each single-trial data at each electrode and time point (and frequency bin for TF analysis) had the general form (1), with being Y the single trial measurement (voltage amplitude for the ERP or relative power for the TFR), *β*_0_ the intercept, *β*_1_ to *β*_4_ the beta coefficients for each experimental condition to be estimated, X_1_ to X_4_ the coding for each column corresponding to the type of stimulus in the design matrix (Figure 2), and *ε* the error term.

Y = *β*_0_ + *β*_1_X_1_ + *β*_2_X_2_ + *β*_3_X_3_ + *β*_4_X_4_ + ε (1)

Then, for the group-level analysis, 2 × 2 repeated measures ANOVAs (generalized Hotelling’s T^2^) were computed on the beta estimates using robust bootstrapping, with the stress pattern and expectancy as within-subject factors. Significant interactions were subsequently resolved using a simple contrast analysis. For all the analyses, a non-parametric bootstrap spatiotemporal clustering approach was used to correct for multiple comparisons, where the family-wise error rate was controlled at an alpha level of 0.05 [52].

## 3. Results

### 3.1. ERPs

**Figure 3:**
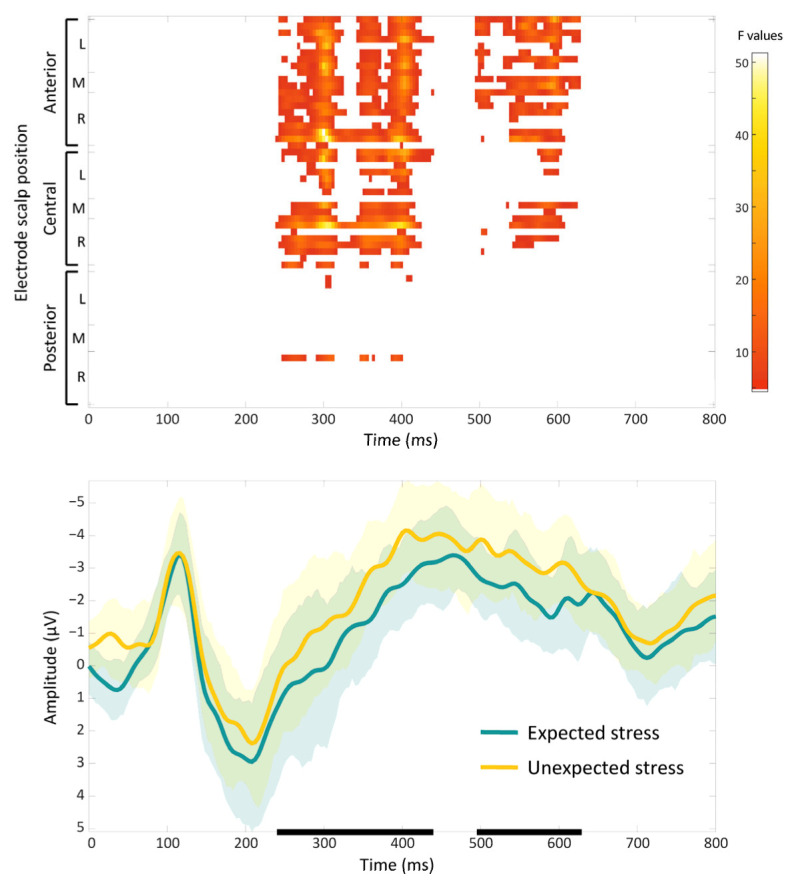
Effects of the stress expectancy on the single-trial event-related potentials (ERPs). (**Top**) Heat map indicating significant F values at *p* < 0.05 across electrodes and time points. The results were corrected with spatiotemporal clustering at an alpha level of 5%. (**Bottom**) Grand average ERP waveforms (20% trimmed mean of participant means) at a representative midline centro-frontal electrode, illustrating the increased negative-going activity for unexpected stress relative to the expected stress in target words. Shaded areas around each condition’s grand average ERP waveform represent the 95% Bayesian Highest Density Interval (HDI). Each bold line on the x-axis indicates the time window of a significant cluster.

The mixed-model ANOVAs revealed a significant main effect of expectancy but no significant main effect of stress or expectancy and stress interaction. The main effect of expectancy was observed in two clusters that covered central and frontal regions and spanned the time windows from 240 to 628 ms (cluster 1: from 240 to 440 ms, maximum F = 51.29, corrected *p* = 0.002; cluster 2: from 496 to 628 ms, maximum F = 30.53, corrected *p* = 0.033). In both clusters, the target words with an unexpected stress pattern elicited a more negative deflection compared with those with an expected pattern (Figure 3).

### 3.2. TFRs

The results of the mixed-model ANOVAs indicated several significant findings associated with the main effects of the expectancy and stress pattern, as well as a significant interaction between these two factors. Given that significant clusters emerged within distinct time windows and frequency bands, the main effects and interaction are detailed separately in the following section.

**Figure 4:**
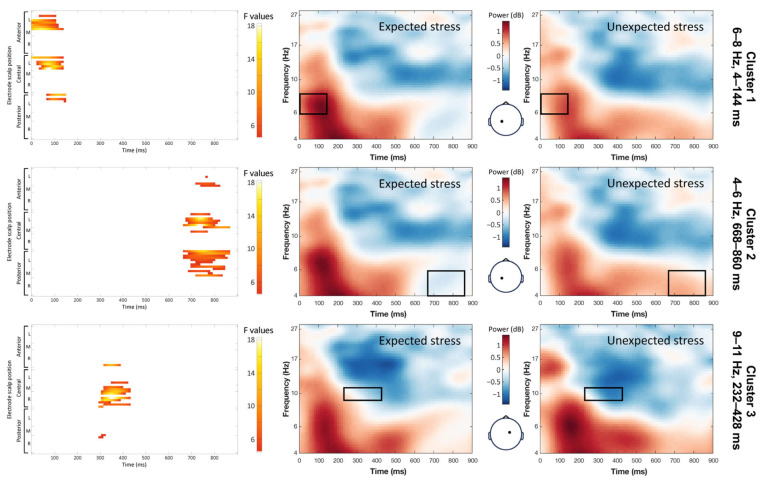
Effect of the stress expectancy on the single-trial time–frequency representations (TFRs). Each row represents a significant cluster identified by the ANOVA. Left: Heat map showing significant F values (*p* < 0.05) across the electrodes and time points (corrected with spatiotemporal clustering at an alpha level of 5%). Middle: Average TFRs for the target words with expected stress. Right: Average TFRs for the target words with unexpected stress. Black rectangles represent the time window and frequency band of the significant cluster.

The main effect of expectancy included significant clusters in the theta and alpha bands (Figure 4). Target words with expected stress patterns showed increased power in the 6–8 Hz frequency range compared with words with unexpected stress patterns (maximum F = 13.96, corrected *p* = 0.012). This effect was observed between 4 and 144 ms after the word onset and localized to the left parietal, central, and frontal scalp regions. Target words with expected stress patterns also showed increased power in the 9–11 Hz range over the right-central and temporal scalp regions 232 to 428 ms post-word onset (maximum F = 9.55, corrected *p* = 0.012). By contrast, target words with unexpected stress patterns displayed higher power in the 4–6 Hz range (maximum F = 18.30, corrected *p* = 0.012). This cluster occurred between 668 and 860 ms post-word onset and was more pronounced over the scalp’s left-central and posterior regions.

**Figure 5:**
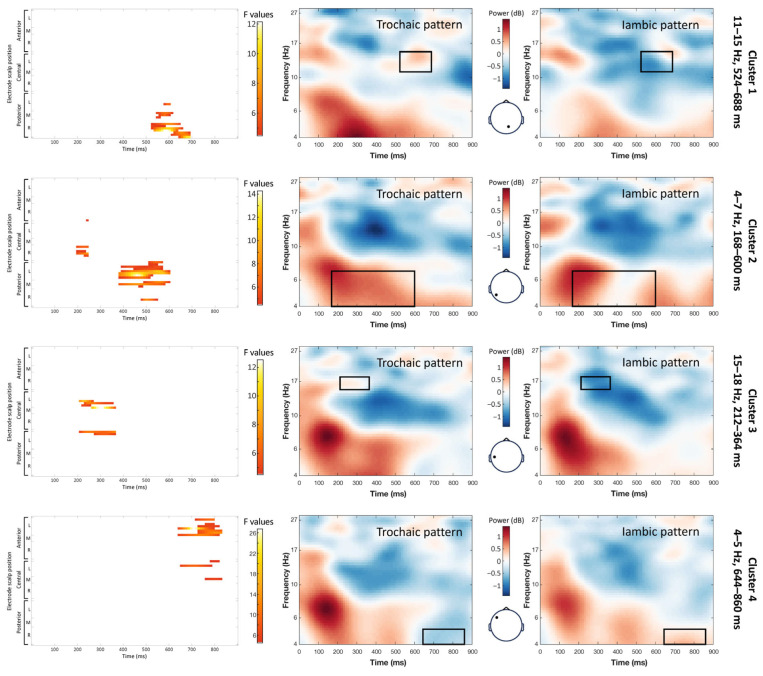
Effect of the stress pattern on the single-trial time–frequency representations (TFRs). Each row represents a significant cluster identified by the ANOVA. Left: Heat map showing the significant F values (*p* < 0.05) across the electrodes and time points (corrected with spatiotemporal clustering at an alpha level of 5%). Middle: Average TFRs for the target words with a trochaic pattern. Right: Average TFRs for the target words with an iambic pattern. Black rectangles represent the time window and frequency band of the significant cluster.

The main effect stress pattern yielded significant clusters in the theta and beta bands (Figure 5). First, a key difference emerged in the 4–7 Hz frequency range from 168 to 600 ms (maximum F = 14.26, corrected *p* = 0.012). Notably, the power was higher for target words with a trochaic pattern compared with those with an iambic pattern that extended from the posterior left to the central-right regions of the scalp. Next, between 212 and 364 ms, there was a significant cluster that resulted from a stronger deactivation in the mid-beta band (15–18 Hz) for iambic rather than trochaic target words over the left parietal regions (maximum F = 12.52, corrected *p* = 0.048). Between 524 and 688 ms, the iambic target words also had a stronger low-beta (11–15 Hz) deactivation than trochaic words over the right parietal and occipital regions (maximum F = 12.14, corrected *p* = 0.012). The last cluster showed a significant difference in the low-theta range (4–5 Hz), between 644 and 860 ms (maximum F = 26.74, corrected *p* = 0.012). In this cluster, the power was higher for iambic than trochaic patterns over the left-central and frontal regions.

**Figure 6:**
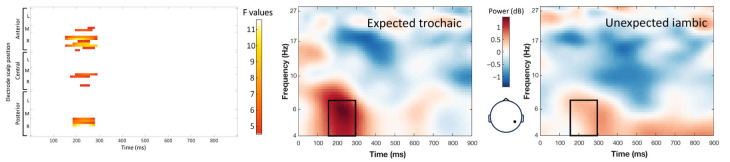
Interaction of the stress expectancy by stress pattern on the single-trial time-frequency representations (TFRs) in the theta band (4–7 Hz). Each row represents a significant cluster identified by the ANOVA. Left: Heat map showing significant F values (*p* < 0.05) across electrodes and time points (corrected with spatiotemporal clustering at an alpha level of 5%). Middle: Average TFRs for the trochaic target words following a trochaic context (i.e., expected trochaic). Right: Average TFRs for the iambic target words following a trochaic context (i.e., unexpected iambic). Black rectangles represent the time window and frequency band of the significant cluster.

**Figure 7:**
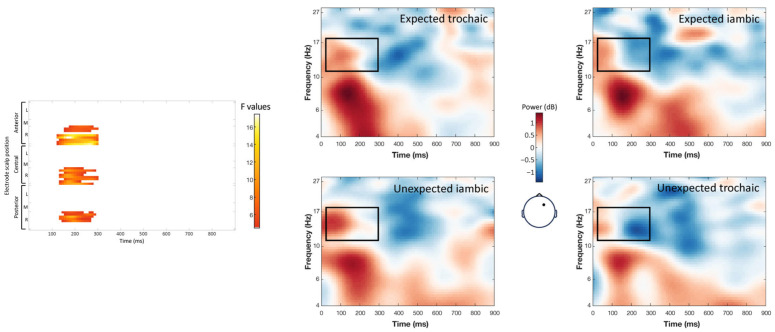
Interaction of the stress expectancy by stress pattern on single-trial time–frequency representations (TFRs) in the low-beta band (11–18 Hz). Each row represents a significant cluster identified by the ANOVA. Left: Heat map showing the significant F values (*p* < 0.05) across electrodes and time points (corrected with spatiotemporal clustering at an alpha level of 5%). Right: Average TFRs for the target words in each of the four experimental conditions.

The interaction between word expectancy and stress pattern elicited two distinct clusters of significant effects. A theta band cluster (4–7 Hz, 156–296 ms; maximum F = 11.62, corrected *p* = 0.012) showed increased power for expected trochaic compared with expected iambic words over a broad right scalp region (Figure 6). A low/mid-beta band cluster (11–18 Hz, 24–296 ms; maximum F = 17.47, corrected *p* = 0.012) revealed increased power for conditions with a preceding trochaic context (regardless of the expectancy) compared with an iambic context over the right-central and frontal regions. All post hoc contrasts were significant at *p* < 0.05 (Figure 7).

## 4. Discussion

In this study, we examined the neural correlates of processing implicit speech rhythm cues (specifically, lexical stress) during silent reading. Utilizing a robust single-trial analysis of ERPs and TFRs, we investigated whether brain responses differentiated between expected and unexpected lexical stress, given a preceding metric context. Additionally, we explored the potential impact of the type of stress pattern on neural activity by comparing the responses to trochaic (more common) and iambic (less common) stress patterns. Our findings revealed several key insights. First, unexpectedly stressed target words, irrespective of the stress pattern, elicited an enhanced negative ERP component followed by increased theta power. Words with the expected trochaic stress pattern elicited early increases in both theta and beta powers, where the trochaic words generally showed a higher theta activity than the iambic words. Notably, this theta difference appeared earlier for expected trochaic words. Additionally, the beta activity was modulated by both the stress pattern and metrical expectancy. The following discussion elaborates on the interpretation of these effects.

### 4.1. Effect of Metrical Expectancy

Consistent with prior ERP research on the perception of overt speech rhythm cues [17,18,20,21,22,24,25,26,27,28], we observed an enhanced negative ERP component for target words with unexpected stress patterns. This effect emerged over the centro-frontal regions within the first 400 ms post-stimulus, mirroring previous findings. Our results also partially converged with the limited studies on implicit speech rhythm, which reported negativities between 250 and 500 ms for unexpected trochaic words [40,42,43].

The negative ERP effect elicited by unexpected stress patterns has been attributed to several distinct, yet not mutually exclusive, mechanisms. It may reflect a contingent negative variation (CNV) generated in anticipation of a stressed syllable, and this is sustained when an unexpected unstressed syllable occurs instead [20,23]. Alternatively, the effect may represent an enhanced N400 component typically associated with lexico-semantic processing, potentially indexing increased semantic processing demands due to the unexpected stress pattern [20,21,23,26]. Finally, the negativity could be a subcomponent of the left anterior negativity (LAN), suggesting a more domain-general error detection mechanism responding to deviations from rule-based predictions [18,22,24,28]. In the context of the present study, the N400 interpretation seems the most compelling, as it is accompanied by a subsequent increase in theta power—a pattern previously associated with semantic processing difficulties in response to unexpected words [53].

It is worth noting that in our study, unexpected stress patterns triggered a heightened negative ERP response, regardless of whether the stress was iambic or trochaic in nature. This finding contrasts somewhat with previous studies on implicit prosody, which showed more varied results in this regard. This variability may stem from several factors, including methodological differences and the complex interplay of cognitive processes involved in implicit prosody processing. For instance, Kriukova and Mani [41] observed an increased positivity for unexpected iambic stress and small but nonsignificant increased negativity to unexpected trochaic stress, potentially due to their weak metrical context and long interstimulus interval (ISI). These factors may have led to a closure positive shift (CPS), masking the expected negativity, particularly for less frequent iambic words. Furthermore, Breen and colleagues [43] used stress-alternating noun–verb homographs embedded in the final position of rhyming couplets. Since lexical stress influences not only acoustic properties (such as alterations in duration, intensity, and pitch) but also phonemic structure (e.g., vowel reduction, consonant clarity), manipulating stress in their study may have disrupted the expected rhyming pattern, introducing confounding phonological factors. These factors highlight the challenge of isolating ERP effects specifically related to metrical expectations when other phonological aspects are simultaneously manipulated. Finally, individual differences in language proficiency and sensitivity to less common metrical structures, like iambic stress patterns in English [1], may contribute to the variability in ERP findings. Previous research supports this notion, linking the amplitude of the negativity evoked by unexpected stress patterns to individual differences in musical rhythm perception skills [27] and reading comprehension ability in adults [54]. In conclusion, our findings contribute to the growing literature on implicit prosody processing while underscoring the intricate interplay of metrical expectations, phonological factors, and individual differences in shaping neural responses to unexpected stress patterns during silent reading.

Mirroring the ERP findings, stress expectancy also influenced the TFRs. Expected stress patterns elicited increased power in the high-theta band (6–8 Hz) over a broad left-parietal to frontal-scalp area. This heightened theta activity aligns with previous research that demonstrated enhanced theta power during the processing of rhythmically regular speech, such as nursery rhymes [55]. Theta activity, especially over the frontal and temporal scalp regions, was shown to synchronize with rhythmic cues in the speech stream, such as syllable boundaries and stress patterns [29]. Recent research suggested that theta activity may play a crucial role in processing linguistic structure and acting as a scaffolding that aids with the integration of incoming linguistic information [30]. Our findings suggest this role may extend to reading. This notion is further supported by evidence of impaired neural tracking of speech rhythm cues in the theta band in individuals with reading deficits [56,57]. Additionally, recent research combining eye tracking and EEG indicated a rhythmic pattern in eye movements during natural reading that is synchronized with specific brain oscillations, notably in the theta band [58]. These findings collectively point to the potential significance of theta band activity in the active processing of individual words during reading.

Finally, words with expected stress patterns also elicited an increase in alpha power. As alpha activity is inversely related to attentional engagement [31], this increase in alpha activity suggests a reduced demand on attentional resources, indicating more efficient processing when word stress patterns match the expectations set by the context.

### 4.2. Effect of Stress Pattern

Both trochaic and iambic words led to increased theta power compared with each other, albeit in different time frames. Notably, the increase in the theta power occurred earlier for trochaic words (with the stress on the first syllable) between 168 and 600 ms, compared with iambic words (with stress on the second syllable), where the increase was seen later between 644 and 860 ms. This finding further suggests that theta activity might be sensitive to stress cues in general, not just a specific type of stress pattern.

In contrast, changes in the beta activity were more selective to the type of stress pattern, with a stronger low-beta deactivation (i.e., a decrease in beta power relative to baseline) found for iambic words than for trochaic words. Prior research linked beta deactivation to semantic processing and lexical access. For instance, stronger beta deactivation was observed for semantically unexpected words during sentence processing and target words following semantically unrelated primes in word pairs [59]. In the context of language comprehension, the modulation of beta activity was proposed to reflect changes in the neurocognitive network underlying the construction of meaning representations, as well as the top-down propagation of the predictions about incoming inputs based on these established representations [60]. In our study, the stronger beta deactivation for iambic words may thus be attributed to their lower prevalence in English [1]. Conversely, the trochaic stress pattern, representing approximately 80% of the lexicon, may facilitate word recognition. Additionally, since trochaic words have initial stress, this effect could also reflect the prioritization of stressed syllables at the beginning of words for quicker access to phonological representations and subsequent lexical processing.

### 4.3. Trochaic Regularity and Beta Oscillations

In the present study, both iambic and trochaic target words were associated with an increase in low-beta activity (11–18 Hz) when following a regular trochaic context, and this effect was independent of their stress expectancy. This finding also aligns with the predictive coding hypothesis described earlier [60], as well as a recently proposed framework for a role of lower beta activity in multimodal temporal predictions [32]. In this regard, increased beta activity could signal the brain’s sensitivity to the established rhythmic structure, aiding the integration of subsequent words into the metrical framework, even if they are unexpected. This may be facilitated by top-down modulation from the metrical framework, where established expectations streamline the processing of incoming words. Additionally, the increased beta activity might reflect enhanced phonological processing due to the clearer rhythmic boundaries provided by the trochaic pattern. Moreover, the stronger beta activity could indicate a heightened attentional focus on words within a trochaic context, potentially due to their salience within the rhythmic structure. Ultimately, the precise interplay of these factors warrants further investigation.

### 4.4. Limitations and Future Directions

While the present study shed light on the neural correlates of implicit stress processing, it did not address individual differences in reading skills. Given the established relationship between speech rhythm perception and reading skills [61,62,63], further study is warranted. As suggested by Fotidzis and colleagues [42], it is possible that individual differences in reading and/or language skills are differentially associated with neural responses to linguistic rhythm during silent reading. Future research investigating the relationship between observed neural markers (e.g., ERP components, theta, delta, and alpha power) and individual variations in reading ability would be informative.

Additionally, future studies should explore the potential relationship between neural tracking of the speech signal (phase alignment of brain oscillations) and the processing of implicit stress patterns. This could provide insights into the underlying mechanisms linking rhythm perception, phonological processing, and reading development, potentially contributing to a deeper understanding of dyslexia and other reading disabilities [57,61].

## 5. Conclusions

This research contributes to our knowledge of the neural responses to lexical stress during a silent reading task. We utilized a conservative, data-driven approach and found the responses varied by the stress expectancy, suggesting lexical stress was a component of the mental phonological representation of words, even when readers’ attention was not drawn to the prosodic features of the text. This refines our understanding of word-level representations produced by readers during silent reading. Furthermore, the similarity of the responses to those in the spoken language literature, both the ERPs and modulations in theta, delta, and alpha bands, suggests overlapping neural networks for the processing of spoken and written language.

## Figures and Tables

**Table 1:** Examples of stimuli in each experimental condition.

Condition	Word 1	Word 2	Word 3	Word 4	Target Word
Expected trochaic	Farmer	Ego	Navy	Cotton	Pencil
Expected iambic	Machine	Giraffe	Cigar	Eclipse	Dispute
Unexpected trochaic	Farmer	Ego	Navy	Cotton	Dispute
Unexpected iambic	Machine	Giraffe	Cigar	Eclipse	Pencil

**Table 2:** Mean log HAL frequency.

Stress Pattern	First Four Words		Target Word	
	Mean	SD	Mean	SD
Trochaic	8.904	1.529	8.941	1.587
Iambic	8.896	1.527	8.914	1.583

Note: HAL—Hyperspace Analogue to Language.

## Data Availability

The raw data supporting the conclusions of this article will be made available by the authors upon request to the corresponding author.
